# An Unusual Case of Choledochoduodenal Fistula Secondary to Peptic Ulcer Presenting With Cholangitis and Pneumobilia

**DOI:** 10.7759/cureus.40915

**Published:** 2023-06-25

**Authors:** Saad AlZahrani, Bayan AlMabadi, Abdulaziz AlZaharani, Mohammed K Shariff

**Affiliations:** 1 Digestive and Liver Center, King Abdullah Medical City, Makkah, SAU; 2 Specialized Surgical Unit, King Abdullah Medical City, Makkah, SAU

**Keywords:** percutaneous transhepatic cholangiography, endoscopic retrograde cholangiopancreatography, cholangitis, duodenal ulcer, choledochodoudenal fistula

## Abstract

Choledochodoudenal fistula is an uncommon bilio-enteric fistula with clinical presentation ranging from having no symptoms to frank cholangitis. The causes of choledochodoudenal fistula are multiple, with bile duct stones being the most common. Duodenal ulcer is rarely the source of choledochodoudenal fistula. Clinical diagnosis defies acumen, and high-quality imaging including endoscopic or radiologic imaging is required for confirmation. Management of choledochodoudenal fistula is not standardized and remains challenging. We report an unusual case of a choledochodoudenal fistula caused by a duodenal ulcer that presented with pneumobilia and cholangitis. Treatment demanded medical, endoscopic, radiologic, and, ultimately, surgical intervention.

## Introduction

Peptic ulcer disease (PUD) is a common disorder. The prevalence of PUD has reduced in recent years. Despite this decline and the advent of proton pump inhibitors, complications of PUD such as bleeding, perforation, obstruction, and, rarely, bilio-enteric fistula remain a healthcare issue [1]. Choledochodoudenal fistula (CDF) in PUD is a rare complication that occurs as a result of a duodenal ulcer penetrating the common bile duct (CBD) [2]. The other possible etiologies of CDF are biliary lithiasis, tumor, Crohn’s disease, tuberculosis, and iatrogenic injury [3-5]. However, sometimes, there may be no obvious cause of this disorder. CDF accounts for 5-25% of all internal biliary fistulas [6]. Progress in hepatobiliary imaging techniques such as endoscopic retrograde cholangiopancreatography (ERCP) and magnetic resonance cholangiopancreatography (MRCP) has resulted in an increase in cases reported over the last 30 years. Here, we describe a case of CDF as a complication of a duodenal ulcer that presented with acute cholangitis and hepatic abscesses.

## Case presentation

A 39-year-old Saudi man on oral hypoglycemic medications for diabetes mellitus and with a history of a duodenal ulcer treated 10 years ago presented to the emergency department with right upper quadrant abdominal pain for four days associated with fever, jaundice, and episodes of vomiting recently ingested food. He was taking non-steroidal anti-inflammatory drugs (NSAIDs) on an intermittent basis for low back pain. On clinical examination, he had a blood pressure of 112/78 mmHg, respiratory rate of 19 breaths/minute, heart rate of 90 beats/minute, oxygen saturation of 95%, and temperature of 38.5°C with icterus and mild right upper quadrant tenderness. Initial laboratory findings revealed leukocytosis, microcytic hypochromic anemia, thrombocytopenia, high inflammatory markers, elevated liver enzymes and bilirubin, and a normal renal function test and coagulation profile (Table 1).

**Table 1:** An overview of the patient’s laboratory workup. ERCP: endoscopic retrograde cholangiopancreatography; PTC: percutaneous transhepatic cholangiography; WBC: white blood cell; HGB: hemoglobin; PLT: platelet; AST: aspartate transaminase; ALT: alanine transaminase; ALP: alkaline phosphatase; INR: international normalized ratio

	Initial	Post-ERCP	Post-PTC insertion	Outpatient clinic follow-up	Normal range
WBC	13.2	12.7	13.5	5.6	3.9–11 × 10^9^/L
HGB	10.8	11.0	10.0	10.3	13.5–17.5 g/dL
PLT	30	77	207	352	150–400 × 10^9^/L
AST	153	105	55	35	8–48 U/L
ALT	306	142	79	26	0–55 U/L
Total bilirubin	10.58	11.7	8.4	2.3	<1.2 mg/dL
Direct bilirubin	8.8	7.8	6.5	1.7	0–0.3 mg/dL
ALP	216	357	310	150	40–150 U/L
INR	1.08	1.12	1.24	1.17	0.9–1.2

**Figure 1:**
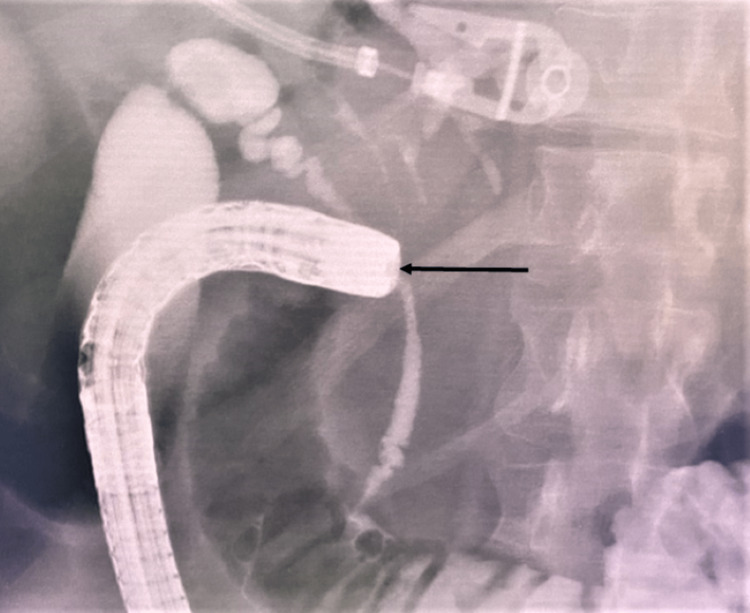
Radiograph showing the passage of the contrast into the biliary tract during injecting the contrast through the fistula.

Two sets of aerobic and anaerobic blood cultures were taken. Ultrasonography demonstrated moderate intrahepatic biliary duct dilation with pneumobilia. Computerized tomography (CT) of the abdomen with contrast showed duodenal wall thickening of the first part reaching 1.4 cm, marked surrounding inflammatory fat stranding, and multiple hepatic abscesses. The CBD was dilated with a thick enhancing wall suggestive of acute cholangitis. There was moderate intrahepatic biliary duct dilatation with pneumobilia within the biliary tree and gallbladder. This necessitated an ERCP that showed a stricture at the duodenal bulb with signs of inflammation and ulcerations. A fistula in the duodenal bulb was noted and contrast injection through the fistula leaked into the CBD, confirming a CDF (Figure 1).

The duodenoscope passed through the stricture with moderate resistance. The major ampulla appeared normal. A cholangiogram obtained by ERCP further confirmed a dilated biliary tree. A 10Fr × 9 cm plastic stent was placed inside the CBD above the level of the CDF to decompress and bridge the defect of the fistula in the biliary tree. Blood cultures grew *Klebsiella pneumonia*, and the patient was started on antibiotics based on sensitivity. In addition, a high-dose proton pump inhibitor was started to treat the duodenal ulcer.

**Figure 2:**
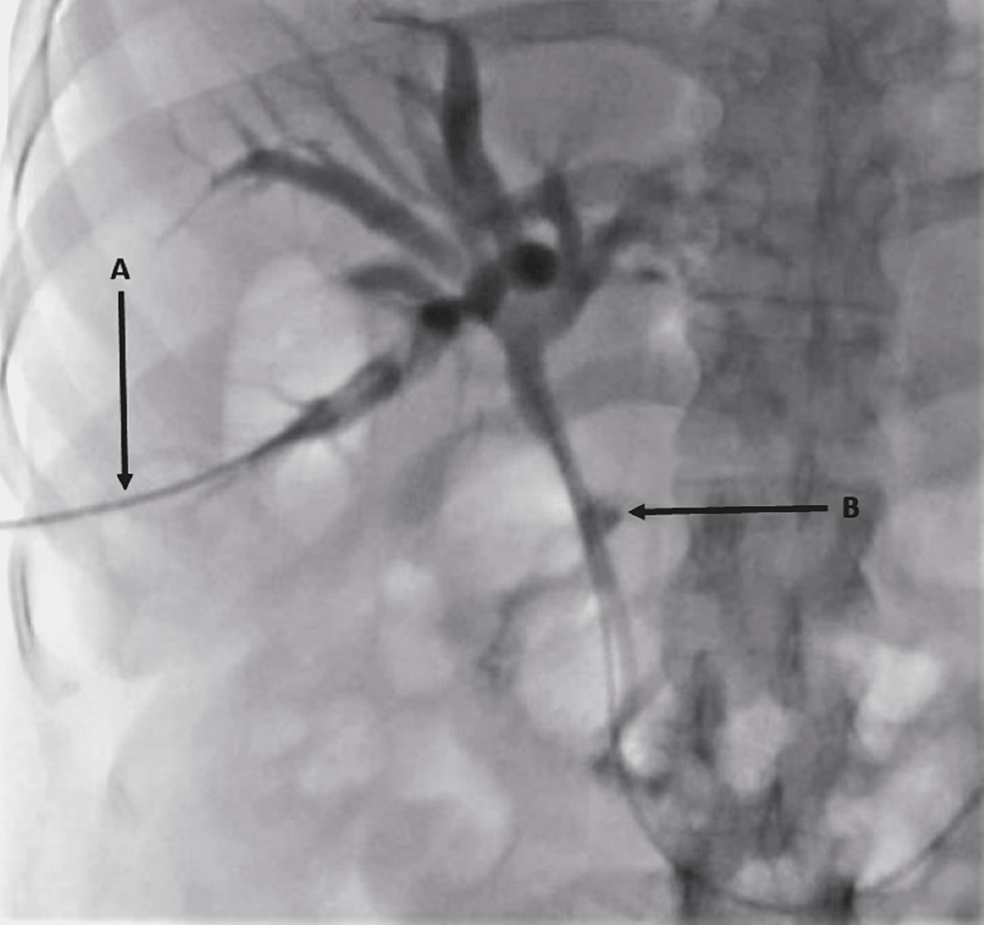
(A) Cholangiogram showing contrast injection by the intervention radiologist through the right intrahepatic ducts, (B) with the passage of the contrast through the common bile duct fistula into the duodenal bulb.

Despite this intervention, the patient showed no signs of clinical or laboratory improvement (Table 1). Because of the duodenal narrowing, percutaneous transhepatic cholangiography (PTC) was performed which showed persistent dilatation of the biliary tree and again confirmed the CDF (Figure 2).

**Figure 3:**
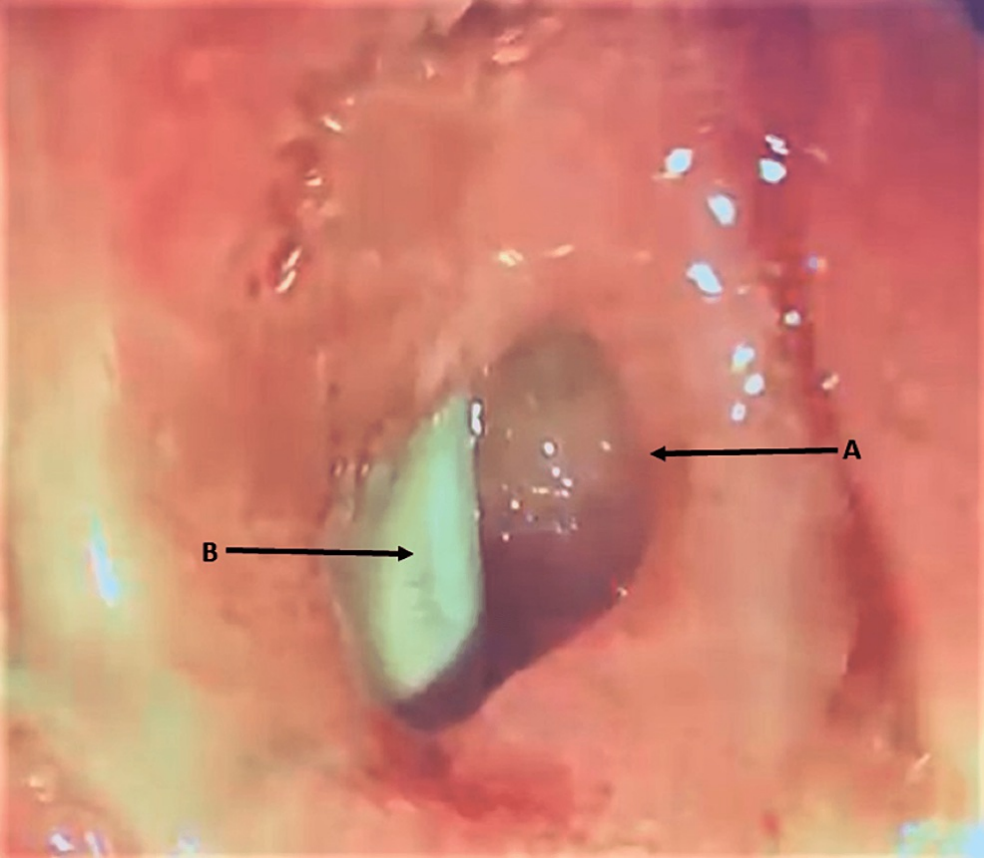
Endoscopic picture of the duodenal bulb, (A) showing the fistula orifice located in the duodenal bulb, penetrating the wall of the bile duct. (B) The biliary plastic stent is visible inside the bile duct.

An internal-external drain was placed to drain the biliary system. An esophagogastroduodenoscopy (EGD) was performed to assess the duodenum, and it showed that the ulceration had improved. However, the CDF orifice in the duodenal bulb had increased in size (Figure 3).

The patient showed no signs of clinical recovery, and following consultation with the surgeon, a decision for surgical intervention into the CDF was made. An exploratory laparotomy with double bypass (Roux-en-Y, gastrojejunostomy, and hepaticojejunostomy) and cholecystectomy were performed. The histopathology of the biopsy taken from the duodenal stricture was consistent with PUD and that of the cystic duct and CBD with chronic active inflammation and ulceration without dysplasia or malignancy. Following the surgery, the patient started to improve and was discharged from the hospital. The patient remained asymptomatic with near normalization of his cell count and liver enzymes when reviewed in the outpatient clinic a month later (Table 1).

## Discussion

Our case was a rare complication of a duodenal ulcer presenting with acute ascending cholangitis. Initial radiological imaging demonstrated pneumobilia and hepatic abscesses. ERCP confirmed the diagnosis of CDF which was further corroborated by the PTC. Biliary drainage at both ERCP and PTC, in addition to medical therapy, did not ameliorate the patient’s clinical condition. This warranted a surgical intervention as a definitive therapy.

The incidence and prevalence of PUD have decreased over the years because of the discovery of *Helicobacter pylori* as the main causative organism and its treatment. However, because of the widespread use of aspirin and NSAIDs, complications of PUD continue to be a significant problem [1]. CDF is a well-recognized but extremely rare complication of PUD [7-10].

CDF could be clinically asymptomatic or present with nonspecific symptoms and may be detected incidentally by gastrointestinal imaging making the clinical diagnosis challenging [2,9,11]. At the other extreme, CDF may manifest as gastrointestinal bleeding and rarely as peritonitis due to a break in the fistulous tract into the abdominal cavity [10,11]. Though typical symptoms are often lacking, most patients with CDF present with right upper quadrant pain, fever, or classical symptoms of cholangitis [2-4]. Our patient presented with the familiar triad of cholangitis, namely, fever, upper quadrant pain, and jaundice.

Diagnosing CDF mandates a high clinical suspicion and needs radiologic or endoscopic imaging to confirm. The presence of pneumobilia on plain X-ray, abdominal ultrasound, or CT scan should raise the suspicion of CDF; however, its presence is not universal [2,3,6,7]. EGD is another modality of investigation that can directly visualize the CDF and establish a cause such as PUD. Nevertheless, if the endoscopist is unaware of CDF, it may be mistaken for a perforation [10]. Cholangiograms at MRCP, ERCP, or PTC are the investigations of choice to confirm CDF and local availability dictates the option. Impediments such as a duodenal deformity or stricture may limit the performance of ERCP, and the absence of intrahepatic dilatation may restrict the use of PTC. Hence, MRCP, which is non-invasive, may be the preferred option in places where it is available. Endoscopic ultrasound is emerging as a favored option for investigating hepatobiliary disorders. Although not yet tested, it may offer an attractive alternative for detecting CDF. Less commonly used tools of diagnosis are barium studies and laparoscopic surgery [2,6,7]. In this case report, our patient was investigated with most of the above-mentioned modalities for both diagnosis and therapy, starting with the detection of pneumobilia on ultrasound and CT which raised the suspicion of CDF, then proceeding to EGD, ERCP, and PTC for confirmation and treatment.

Because of its rarity, CDF management is not standardized and remains a challenge. Most available treatment options are established by small retrospective studies or case reports and series [2,4,8,11]. In general, management is dictated by the patient’s presentation; the presence of complications; and the type, size, and etiology of the fistula. A proposed treatment algorithm would be initial medical management to relieve symptoms and allow healing of the fistula, especially in cases of ulcer disease. Depending on clinical manifestation, this may be followed up in cases of failure or may simultaneously require endoscopic therapy. For example, patients presenting with cholangitis will need ERCP, and depending on the finding, may require bile duct stone extraction or balloon dilatation for biliary stenosis, in addition to biliary stent placement. Failing this, surgery becomes imperative with options including resection of involved segments such as the stomach, duodenum, biliary tree, and gallbladder; biliary reconstruction or stricturoplasty; and fistula repair with biliary enteric anastomosis [2,4]. Others have advocated that therapy be based on the CDF size, suggesting that fistulas with an orifice less than 0.5 cm should be managed medically, those between 0.5 and 1 cm should undergo successful drainage of the biliary system, and those greater than 1 cm be should be considered for surgery [4]. We followed the proposed stepwise treatment algorithm mentioned above rather than the one based on CDF size. In our case, we carefully assessed the patient at each step and moved to the next therapy in line, to ultimately offer the definitive curative treatment which was an exploratory laparotomy with double bypass (Roux-en-Y, gastrojejunostomy, and hepaticojejunostomy) with cholecystectomy. This stabilized our patient’s condition, enabling his discharge from the hospital and facilitating a healthy state when eventually reviewed in the clinic.

## Conclusions

Choledochoduodenal fistula is a rare complication of a duodenal ulcer. Diagnosis warrants awareness and a high clinical suspicion. A range of investigative modalities can be used for confirmation depending on availability and expertise. Management lacks consensus and varies including medical, endoscopic, and surgical options. This case report raises awareness of this uncommon disorder and demonstrates the use of different modalities in the diagnostic workup. In addition, it illustrates the benefit of an algorithm-based management strategy to achieve conclusive treatment.
